# The Influence of Potassium Hexafluorophosphate on the Morphology and Anticorrosive Properties of Conversion Coatings Formed on the AM50 Magnesium Alloy by Plasma Electrolytic Oxidation

**DOI:** 10.3390/ma16247573

**Published:** 2023-12-09

**Authors:** Łukasz Florczak, Barbara Kościelniak, Agnieszka Kramek, Andrzej Sobkowiak

**Affiliations:** 1 Department of Physical Chemistry, Faculty of Chemistry, Rzeszow University of Technology, 35-959 Rzeszow, Poland; 2 Department of Materials Science, Faculty of Mechanical Engineering and Aeronautics, Rzeszow University of Technology, 35-959 Rzeszow, Poland; b.koscielnia@prz.edu.pl; 3 Department of Component Manufacturing and Production Organization, Faculty of Mechanics and Technology, Rzeszow University of Technology, 37-450 Stalowa Wola, Poland; a.kramek@prz.edu.pl

**Keywords:** magnesium alloy, plasma electrolytic oxidation, corrosion resistance, microstructure

## Abstract

In this study, conversion coatings were produced on the AM50 magnesium alloy by a plasma electrolytic oxidation (PEO) process in alkaline-silicate electrolyte with the addition of potassium hexafluorophosphate, using a unipolar pulse power source. The coating microstructure and its composition were determined using scanning electron microscopy (SEM) and an X-ray photoelectron spectroscopy (XPS). The corrosion resistance of the conversion coatings was evaluated by means of potentiodynamic polarization tests (PDP) and electrochemical impedance spectroscopy (EIS) in a dilute Harrison solution (DHS). It has been found that the properties (microstructure, composition, and coating thickness) of the obtained layer and, therefore, their anticorrosive resistance strongly depend on the electrolyte composition. The best anticorrosive properties were observed in the layers obtained in the presence of 2.5 g/L KPF_6_. It was found that the conversion coating produced with the addition of hexafluorophosphate is characterized by a different morphology (sponge-like) and better anticorrosion properties, in comparison to the coating obtained with the addition of fluoride and orthophosphate salts commonly used in PEO synthesis. The sponge-like structure, which is similar to bone structure in combination with the presence of phosphates in the layer, can increase the biocompatibility and the possibility of self-healing of this coating. However, neither Mg(PF_6_)_2_, nor any other compounds containing PF_6_^−^, have been found in the layers produced.

## 1. Introduction

Magnesium is the lightest construction material (1.74 g/mL), but due to its high reactivity and poor mechanical properties, it is practically not used in its pure form. The most frequently added alloying element is aluminum, which ensures high strength, creep resistance, and anticorrosive properties. The addition of zinc increases the ductility and castability, and improves the strength of the alloy at room temperature. Manganese increases the strength of the alloy, enables its weldability, and increases the hardness of Mg-Al alloys. Calcium primarily increases the biocompatibility of alloys (accelerates bone growth), improves the mechanical properties, and the anticorrosive properties. The addition of rare earth elements, for example, Ce, La, Nd, and Gd, improves the mechanical properties at elevated temperatures, and the resistance to corrosion [1,2].

Magnesium alloys, due to their excellent properties—such as a high strength to weight ratio, good dimensional stability, electromagnetic shielding, and biocompatibility—are widely used in industry. The most commonly used alloys include those with the addition of aluminum and zinc or manganese (AZ and AM series), as well as rare earth elements (RE). Alloys such as AZ91, AM50, or WE43 are used in the electronic, automotive, and aerospace industries [3,4,5,6]. Magnesium-calcium alloys can be used in biomedical applications, e.g., biodegradable orthopedic implants [7,8]. However, magnesium alloys are characterized by very low corrosion resistance, which is caused by the high chemical activity of magnesium, and also by the unstable passive layer formed on the surface of the alloys [2,9,10,11]. To improve the corrosion resistance of magnesium alloys, an appropriate surface treatment is necessary to produce anticorrosive coatings on the substrate [12,13]. Plasma electrolytic oxidation (PEO) is a useful technique for developing a protective coating on magnesium alloys, which involves generating sparks on the alloy surface to produce relatively thick, dense, and hard ceramic oxide coatings [14,15,16,17,18,19,20].

This is a high-voltage anodization process, in which metallic magnesium, acting as the anode of the system, is oxidized to Mg^2+^, which reacts with components of the electrolyte. Since strongly alkaline baths (pH~13) are used in the process the most often, the intermediate product is magnesium hydroxide [Mg(OH)_2_], which, as a result of the high process temperature (approximately 3000 K), is immediately dehydrated to form magnesium oxide (MgO), the main end product of the PEO process. When using additives in the electrolytic bath, such as silicates, phosphates, aluminates, or fluorides, appropriate magnesium salts are also incorporated into the coating structure [17]. The formation of the conversion coating in the PEO process occurs simultaneously with the intensive evolution of gases (oxygen and hydrogen), and therefore the overall process is the sum of electrochemical processes, plasma chemical reactions, and thermal diffusion of oxygen [20].

The most commonly used types of baths in the PEO process include silicate [21,22] and phosphate electrolytes [23,24], which ensure appropriate mechanical and anticorrosive properties of the coatings are produced [25,26]. Baths combining both components are also used to further optimize the protective properties of the synthesized surface layers. The presence of silicate increases the hardness of coatings, and phosphates improve biocompatibility and biodegradability, which is particularly important in biomedical applications of magnesium alloys [27,28]. In the case of phosphates, the properties of coatings depend not only on their amount, but also on the kind of compound used, for example: Na_3_PO_4_ [23,24,29], Na_2_HPO_4_ [30], (NaPO_3_)_6_ [28,31], and Na_4_P_2_O_7_ [32]. Another important ingredient often added to PEO baths is fluoride, which increases the corrosion resistance, surface hardness, and wear resistance of the protective layer [33,34,35]. Simple fluorides (NaF [33], KF [34], or CaF_2_ [36]) are used the most frequently, but attempts have also been made to use substances containing multifluoride anions, for example K_2_ZrF_6_ [37,38,39], K_2_TiF_6_ [40,41], NaSiF_6_ [42,43], Na_3_AlF_6_ [44,45], and NaBF_4_ [46].

In this study, the influence of the addition of KPF_6_ to electrolytes on the structure and anticorrosive properties of PEO coatings produced on the AM50 magnesium alloy has been investigated. To our knowledge, the application of KPF_6_ to electrolytic baths has not yet been reported. The analysis of the impact of the addition of KPF_6_ on the PEO process is interesting because there are reports in the literature that state that the PF_6_^−^ ion is not hydrolyzed in a strongly alkaline medium (pH > 12) [47], unlike K_2_ZrF_6_ [39], K_2_TiF_6_ [41], or Na_3_AlF_6_ [44]. Therefore, the formation of a polyfluorine magnesium salt in the structure of the conversion coating is possible, as we found in the case of the addition of NaBF_4_ salt [46]. The properties of conversion coatings prepared at the optimal concentration of hexafluorophosphate ions have also been compared with those produced in a bath containing equimolar amounts of fluorine and phosphorus in the form of the most commonly used fluoride and orthophosphate salts.

## 2. Materials and Methods

### 2.1. Materials and Coatings Preparation

AM50 magnesium alloy (Neo Cast, Krakow, Poland), with the composition presented in Table 1, was used as substrate for PEO treatment. Before PEO, the specimens in the shape of rectangular plates of 50 mm × 50 mm × 8 mm were polished with SiC abrasive paper to a grit of 1200. The samples were then washed in deionized water, ultrasonically degreased with acetone, and dried. Subsequently, they were electrochemically activated as anodes in a saturated NaF solution for 5 min at a voltage equal to 80 V (DC). Immediately after this procedure, the sample was rinsed with deionized water and submitted to the PEO process.

The electrolyte used in the PEO process consists of NaOH (4 g/L) and Na_2_SiO_3_·5H_2_O (10 g/L). The coatings were prepared in the electrolyte without and with KPF_6_ in an amount ranging from 0.5 to 4.0 g/L. Additionally, the synthesis was carried out in a bath containing a mixture of NaF and Na_3_PO_4_, instead of KPF_6_. All solutions were prepared using commercially available, analytical grade reagents and deionized water (conductivity below 0.1 μS/cm at 25 °C). The PEO process was performed using a pulse electrical source pe861UA-500-10-24-S (Platnig Electronic GmbH, Sexau, Germany). The electrical parameters were set as follows: frequency 200 Hz, duty cycle 40%, and current density 5 A/dm^2^. The PEO process time was 10 min and the electrolyte temperature was kept within the 5–15 °C range. The magnesium alloy sample was used as an anode, and stainless steel as a cathode. The coatings obtained were cleaned with deionized water, and dried.

### 2.2. Coatings Characterization

The thickness of the coatings was measured using a Dualscope MP20 eddy current film thickness measurement gauge (Fischer, Sindelfingen, Germany) with FTA 3.3 H sonde. The average layer thickness and standard derivation of each sample were calculated from 20 measurements.

The surface roughness of the coated surface was measured using a Surftest SJ-210 (Mitutoyo, Kawasaki, Japan). The standard roughness parameter (*R*_a_) as the arithmetic mean deviation was determined based on measurements performed 10 times in a row.

A scanning electron microscope (SEM) Phenom XL (Thermo Fisher Scientific Inc., Waltham, MA, USA) was used to characterize the surface and cross-sectional morphology of the PEO coatings.

An energy dispersive X-ray spectrometer (EDS) attached to an SEM was employed to analyze the elemental distribution in the coating. Composition of outer and inner layer PEO coating was investigated by an X-ray Photoelectron Spectrometer (XPS) using a K-Alpha anode (Thermo Fisher Scientific Inc., Waltham, MA, USA), equipped with an argon ion gun to obtain measurements in the inner layer. All energy values were corrected according to the adventitious C 1s set at 284.5 eV.

Electrochemical corrosion tests were carried out using a PARSTAT 2273 (Princeton Applied Research, Houston, TX, USA) potentiostat in a conventional three-electrode cell with the sample as the working electrode (exposed area 0.785 cm^2^), a platinum plate as the auxiliary electrode, and a silver chloride electrode immersed directly in a corrosive solution as the reference electrode. The corrosion resistance of the samples was evaluated by using potentiodynamic polarization (PDP) curves and electrochemical impedance spectroscopy (EIS) measurements in a dilute Harrison solution [0.35 wt.% (NH_4_)_2_SO_4_, 0.05 wt.% NaCl] at room temperature (22 ± 1 °C). The potentiodynamic polarization curves were measured from −0.25 to +0.25 V with respect to the open circuit potential (OCP) at a scan rate of 1 mV/s after an initial 3 h exposure in solution to stabilize OCP. The Tafel analysis of the potentiodynamic curves was performed, and the values of the following electrochemical parameters were obtained: corrosion potential (*E*_CORR_), corrosion current density (*j*_CORR_), and anodic (*β*_A_) and cathodic (*β*_C_) Tafel slopes. Polarization resistance (*R*_P_) was calculated according to the Stern-Geary equation [49]:

(1) $$R_{P}=\frac{\beta_{A}\cdot \beta_{c}}{2.303\cdot j_{\mathrm{CORR}}\left(\beta_{A}+\beta_{C}\right)}$$

EIS measurements were conducted at AC frequencies ranging from 100 kHz to 10 mHz at an interval of 10 points per decade, with 10 mV rms after an initial 3 h exposure in solution. The results obtained were analyzed using an equivalent circuit, which was found by the fitting procedure implemented in the ZSimpWin 3.21 software (EChem Software, Ann Arbor, MI, USA).

## 3. Results and Discussion

### 3.1. Plasma Electrolytic Oxidation Process

**Figure 1:**
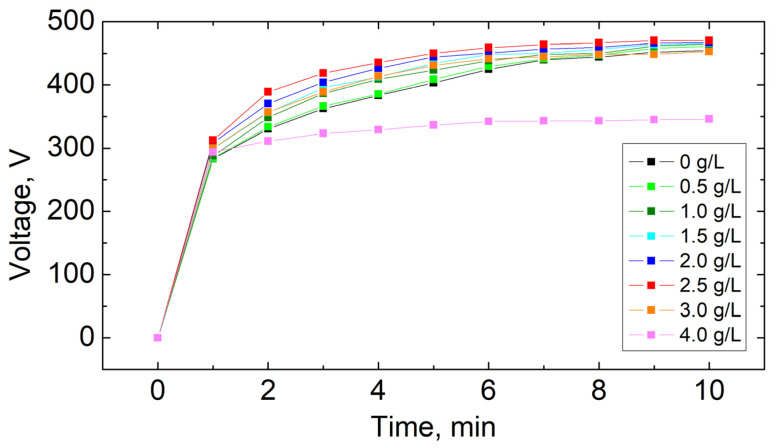
Voltage-time curves during the PEO process in the electrolyte containing different concentrations of KPF_6_.

**Figure 2:**
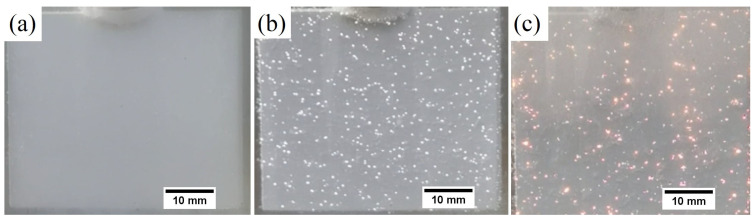
Images of the magnesium alloy surface during the PEO process at its various stages: (**a**) conventional anodization, (**b**) spark anodization, and (**c**) micro-arc oxidation.

A voltage-time curve was used to investigate the influence of electrolyte composition on the PEO process (Figure 1). Three typical stages were identified during the PEO process, which included conventional anodization (without discharges), spark anodization (with small white discharges), and micro-arc oxidation (with large orange discharges). Figure 2 presents images of samples at particular stages. These stages are separated by characteristic voltage values called breakdown voltage and critical voltage. Based on observations of the surface of magnesium alloy samples during the PEO process, it was found that the composition of the electrolyte did not noticeably affect the value of breakdown voltage and critical voltage, which were approximately equal to 200 V and 450 V, respectively. The time for the first visible sparks to appear, which means that the breakdown voltage was reached, was approximately 30 s, regardless of the amount of KPF_6_ in the solution. Table 2 presents the time values needed to reach the critical voltage (450 V) and the final voltage values for different concentrations of KPF_6_ in the electrolyte.

**Figure 3:**
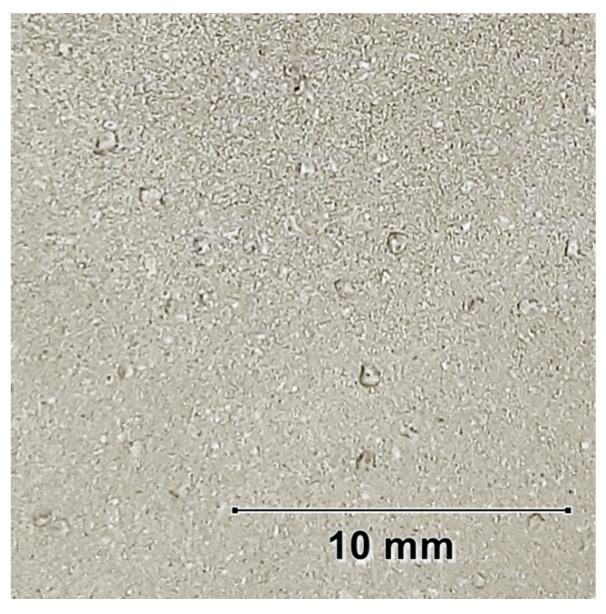
Image of the magnesium alloy surface after the PEO process in an electrolyte 4 g/L KPF_6_.

The increase in KPF_6_ concentration in the electrolyte up to 2.5 g/L decreases the time to reach the critical voltage from 8.8 to 5.0 min. In these cases, the obtained coatings are smooth, continuous, and without visible damage. Higher KPF_6_ content causes the time to increase, and for 4 g/L the critical voltage is not achieved. The final voltage values also increase with the addition of KPF_6_ up to 2.5 g/L (from 455 to 470 V), and then decrease to 346 V for KPF_6_ concentration equal to 4 g/L addition. When the critical voltage is not achieved, the oxidation process is unstable, with visible local burns resulting from the formation of local pits on the surface of the magnesium sample after the PEO process. (Figure 3).

### 3.2. Thickness and Roughness of PEO Coatings

**Figure 4:**
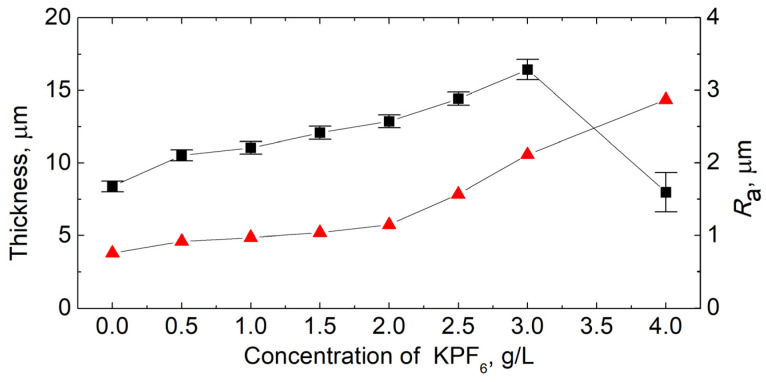
Average layer thickness (black squares) and surface roughness coefficient, *R*_a_ (red triangles) of PEO coatings produced in electrolytes with different concentrations of KPF_6_.

The effect of KPF_6_ concentration on the average thickness and surface roughness of PEO coatings formed on the magnesium alloy is shown in Figure 4.

The measured thickness and roughness coefficient (*R*_a_) of the conversion coating produced by the PEO process in the base bath (without KPF_6_ content) were 8.4 and 0.76 µm, respectively. The introduction of KPF_6_ into the electrolyte in an amount of up to 3 g/L increases the thickness of the coating to 16.4 μm, simultaneously increasing its roughness coefficient to 2.11 μm. The addition of a larger amount of KPF_6_ to the electrolytic bath causes a decrease in the average coating thickness, but also a further increase in its roughness. The coating synthesized in an electrolyte containing 4 g/L KPF_6_ is characterized by a lower average thickness (8.0 µm) than that produced in the base solution. An increase in the standard deviation value in thickness measurements (from 0.4 to 1.4 µm) and an increase in the roughness coefficient (up to 2.9 µm) means that a heterogeneous porous layer with a more variable structure is formed. This suggests that partial dissolution of the coating can occur.

### 3.3. Corrosion Resistance of PEO Coatings

**Figure 5:**
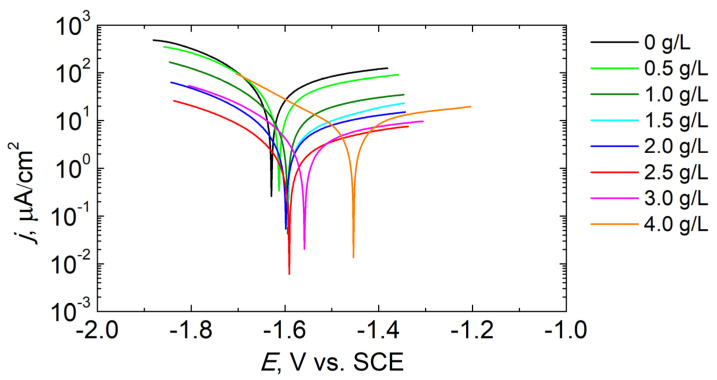
Potentiodynamic curves of PEO coatings synthesized in electrolytes with different concentrations of KPF_6_ after 3 h exposure in DHS.

The anticorrosive performance of all PEO coatings has been evaluated by potentiodynamic polarization (PDP) testing after 3 h immersion in dilute Harrison solution (DHS). The results are shown in Figure 5 and Table 3.

Higher polarization resistance (*R*_P_) and lower corrosion current density (*j*_CORR_) indicate better corrosion resistance. Based on the recorded curves, it was found that the addition of KPF_6_ up to 2.5 g/L systematically decreased *j*_CORR_ (from 33.01 to 1.76 µA/cm^2^) and increased *R*_P_ (from 1.04 to 21.09 kΩ∙cm^2^). The addition of a larger amount of KPF_6_ reverses the observed trend and reduces the anticorrosive properties of PEO coatings. The corrosion potential (*E*_CORR_) shifts to more positive values in the entire tested range of KPF_6_ concentrations in the electrolyte bath.

**Figure 6:**
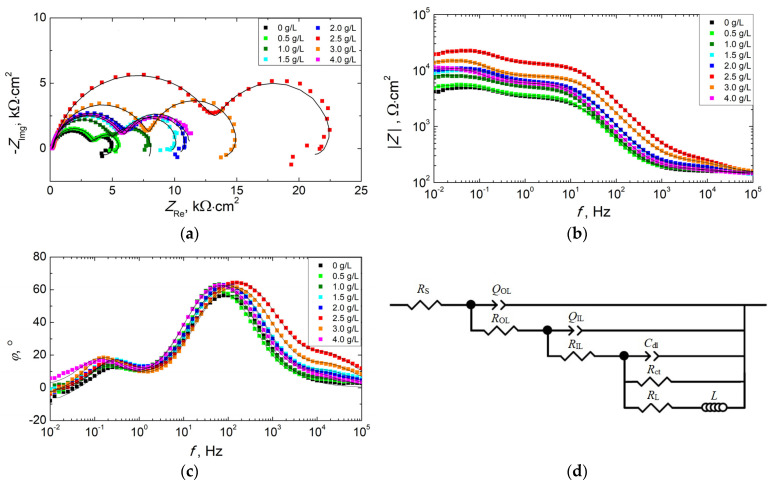
Results of EIS measurements for PEO coatings synthesized in electrolytes with different concentrations of KPF_6_ after 3 h exposure in DHS: (**a**) Nyquist plots, (**b**,**c**) Bode plots. Dots represent experimental data, and lines are the results of the fitting. (**d**) Equivalent circuit model used to fit EIS data measurements of PEO coatings.

To better describe the electrochemical behavior of PEO coatings synthesized in electrolytes with different concentrations of KPF_6_, electrochemical impedance spectroscopy (EIS) studies have been performed. To achieve the best fit of the data obtained, several equivalent circuits were analyzed, taking into consideration the physical model of the conversion coating and the possible corrosion processes. Finally, the impedance data obtained by EIS (Figure 6a–c) were analyzed using an equivalent circuit (EQC) model, as shown in Figure 6d.

In the circuit selected, *R*_S_ represents the resistance of the electrolyte between the working and reference electrodes. Two consecutive groups of parallel combinations of resistors (*R*_OL_ and *R*_IL_) and constant phase elements (*Q*_OL_ and *Q*_IL_) were applied to describe the resistance and capacitance of the outer porous layer (10^3^–10^5^ Hz), and the inner barrier layer (10^3^–10^1^ Hz), respectively. *R*_ct_ and *C*_dl_ represent the resistance of charge transfer and the electrochemical double layer capacitance at the substrate/coating interface, which corresponds to the low frequency time constant (10^1^–10^−1^ Hz). An inductive loop in the low-frequency range is related to the dissolution of Mg and indicates that pitting corrosion of the substrate takes place. It is represented by the resistance *R*_L_ and the inductance *L* [50]. Constant phase elements (*Q*) used in equivalent circuits were selected instead of pure capacitance for better fitting because the surface of the conversion coatings is physicochemically inhomogeneous, uneven, and rough. The *Q* impedance is described as:

(2) $$Z_{\mathrm{CPE}}=\frac{1}{Q_{0}{\left(j\omega \right)}^{n}}$$

where *ω* is the angular frequency, *j* is the imaginary number, *Q*_0_ is the constant admittance, and *n* is the empirical exponent.

The simulated values (fitting parameters) obtained by matching the theoretical model and the experimental data are presented in Table 4. Good fit quality was achieved, which is demonstrated by the low values of the chi-square test (Χ^2^) and the good agreement between the experimental data and the fitting results (dots and lines in Figure 6a–c).

It is possible to calculate the total resistance (*R*_total_) of PEO coatings from the difference between the resistance at low frequency (|*Z*|_*f*→0_) and the resistance of the electrolyte solution (|*Z*|*_f→∞_*) [51,52]. When the frequency goes to zero, the impedance of the capacitive components approaches infinity and the inductive component tends to zero (|*Z_L_*|→0). Therefore, the total resistance of the corrosion system can be calculated from a combination of *R*_OL_, *R*_IL_, *R*_ct_, and *R*_L_:

(3) $$R_{\mathrm{total}}={\left|Z\right|}_{f\to 0}-{\left|Z\right|}_{f\to \infty }=R_{\mathrm{OL}}+R_{\mathrm{IL}}+{\left(\frac{1}{R_{\mathrm{ct}}}+\frac{1}{R_{L}}\right)}^{-1}$$

From the EIS measurements, it follows that after 3 h of immersion in a corrosive medium, the outer layer of the PEO coating has no influence on the anticorrosive properties of the system, and the resistance of the layer is below approximately 0.2 kΩ·cm^2^. The corrosion resistance of the PEO coatings depends mainly on the resistance of the dense inner layer, and the resistance of charge transfer at the substrate/coating interface [23,31,33,53]. With an increase in the amount of KPF_6_ in the bath, an increase in the ratio of *R*_ct_ to *R*_IL_ is observed, which suggests the influence of the additive on the self-healing processes. The sealing process of PEO coatings on magnesium alloys by corrosion products has been reported [39,53]. However, the presence of aggressive ions in the corrosive medium causes pitting corrosion (dissolution of Mg), which is indicated by the inductive loop in the EIS spectrum at low frequencies [50,51,54]. The addition of up to 2.5 g/L KPF_6_ to the electrolyte bath increases the corrosion resistance of the PEO coatings, while its larger amounts result in a reduction in the protective properties of the coatings. The best anticorrosive properties were demonstrated by the PEO coating prepared with the addition of 2.5 g/L KPF_6_ (*R*_total_ = 20.61 kΩ·cm^2^). The observed decrease in the anticorrosive properties of the coatings obtained at higher concentrations is probably due to the hydrolysis of KPF_6_ during the PEO process, with the release of HF. The hydrolysis process of KPF_6_ leading to the formation of HF has been reported [55,56]. An increase in the concentration of KPF_6_ in electrolytic baths can induce the local appearance of larger amounts of HF in the reaction environment, which can cause damage to the formed coatings.

**Figure 7:**
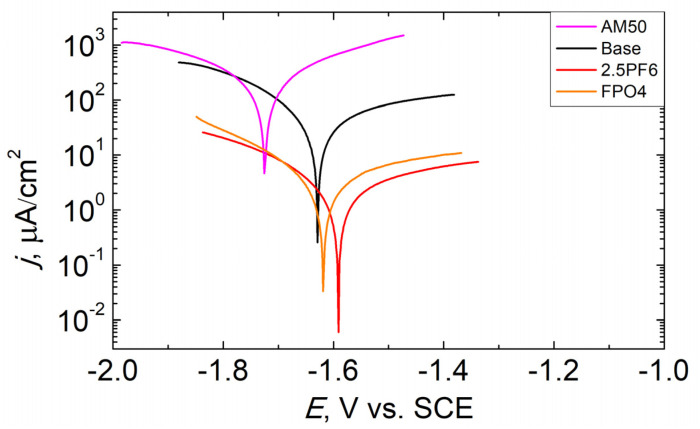
Potentiodynamic curves of the uncoated AM50 alloy and the PEO coatings synthesized in different electrolytes after 3 h exposure in DHS.

**Figure 8:**
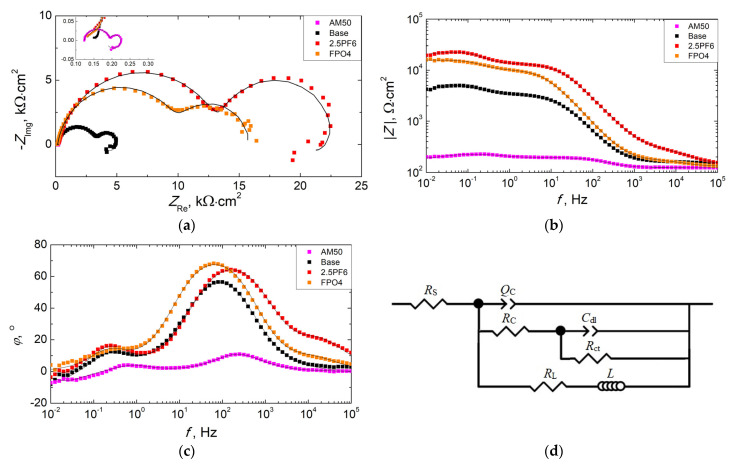
Results of EIS measurements for the uncoated AM50 alloy and PEO coatings synthesized in different electrolytes after 3 h exposure in DHS: (**a**) Nyquist plots, (**b**,**c**) Bode plots. Dots represent experimental data, and lines are the results of the fitting. (**d**) Equivalent circuit model used to fit EIS data measurements of the uncoated AM50 alloy.

To show the advantage of using KPF_6_ instead of the combination of NaF and Na_3_PO_4_, which are commonly applied as additives to the PEO electrolyte baths, the anticorrosive properties of the layers obtained in different electrolytes, listed in Table 5, have been compared. In the baths containing fluorine and phosphorus, their contents were equimolar. The bath containing 2.5 g/L KPF_6_ was chosen for comparison, as the coatings obtained in this electrolyte have shown the best anticorrosive properties. The results of the measurements of potentiodynamic polarization and electrochemical impedance spectroscopy for the coatings obtained, and also for the uncoated AM50 magnesium, are presented in Figure 7 and Figure 8, as well as Table 6 and Table 7.

The uncoated AM50 magnesium alloy shows a very high corrosion rate in the DHS solution (corrosion current density and polarization resistance are equal to 140 μA/cm^2^ and 220 Ω·cm^2^, respectively). The PEO treatment in the base electrolyte increases the corrosion resistance of the substrate by more than 4 times. The introduction of additives containing fluorine and phosphorus into the electrolyte further increases the anticorrosive properties of conversion coatings, and the presence of an additive in the form of a single salt (KPF_6_) is more effective than a mixture of fluoride and orthophosphate (*j*_CORR_ and *R*_P_ are equal to 1.76 μA/cm^2^, 21.09 kΩ·cm^2^ and 2.75 μA/cm^2^, and 13.41 kΩ·cm^2^, for 2.5PF6 and FPO4 samples, respectively).

The impedance spectrum obtained for the coating produced on the FPO4 sample (with NaF and Na_3_PO_4_) does not show a visible induction loop at low frequencies. This means that the value of the total resistance (*R*_total_) is equal to the sum of *R*_OL_, *R*_IL_, and *R*_ct_*)*. In the case of the EIS spectrum of the uncoated AM50 alloy, the occurrence of two capacitive loops (at high and medium frequencies) and an impedance loop at low frequencies can be observed. The first capacitive loop is the result of a naturally formed oxide layer in an aqueous environment (*R*_C_, *Q*_C_), while the second loop can be associated with the charge-transfer process (*R*_ct_, *C*_dl_). The visible inductive loop indicates the presence of localized corrosion (*R*_L_, *Q*_L_) [52]. The equivalent circuit model used to fit the EIS data for measurements on the uncoated magnesium alloy is shown in Figure 8d. The total resistance (*R*_total_) in this case could be obtained by a combination of *R*_C_, *R*_ct_, and *R*_L_:

(4) $$R_{\mathrm{total}}={\left(\frac{1}{R_{C}+R_{\mathrm{ct}}}+\frac{1}{R_{L}}\right)}^{-1}$$

The results obtained have shown that the conversion coatings produced in the PEO process significantly increase the corrosion resistance of the AM50 magnesium alloy (*R*_total_ = 3.8 kΩ·cm^2^). The introduction of additives into the silicate base bath further improves the barrier properties of the coatings. The addition of KPF_6_ makes it possible to obtain better anticorrosive properties of the synthesized coating, compared to a mixture of NaF and Na_3_PO_4_ with an equimolar content of fluorine and phosphorus (the total resistance for the 2.5PF6 sample is higher, compared to the FPO4 sample).

### 3.4. Morphological and Composition Characteristics of PEO Coatings

**Figure 9:**
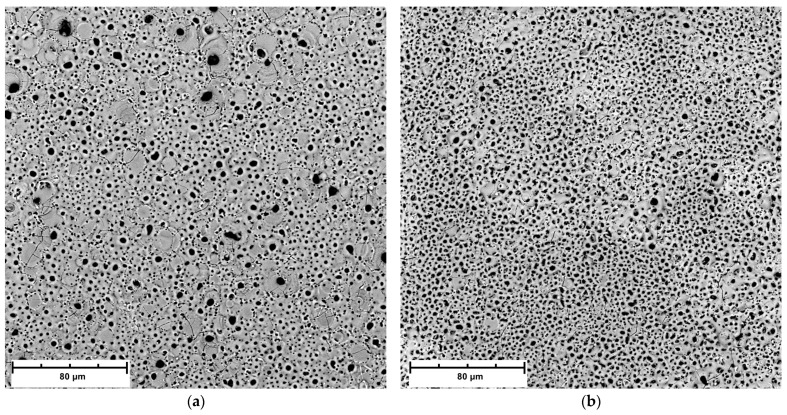

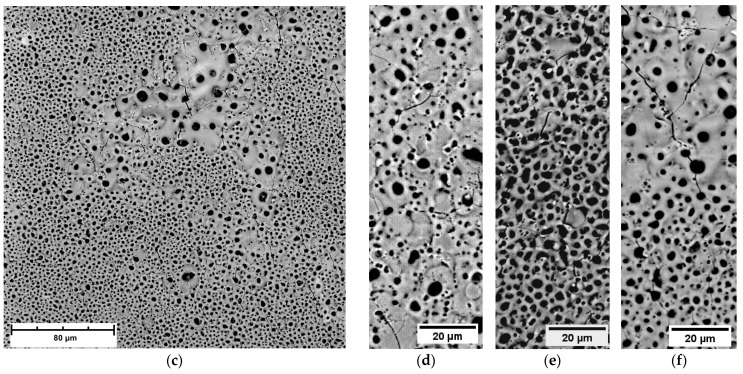
Surface morphology of PEO coatings for different samples: (**a**,**d**) Base, (**b**,**e**) 2.5PF6, (**c**,**f**) FPO4.

**Figure 10:**
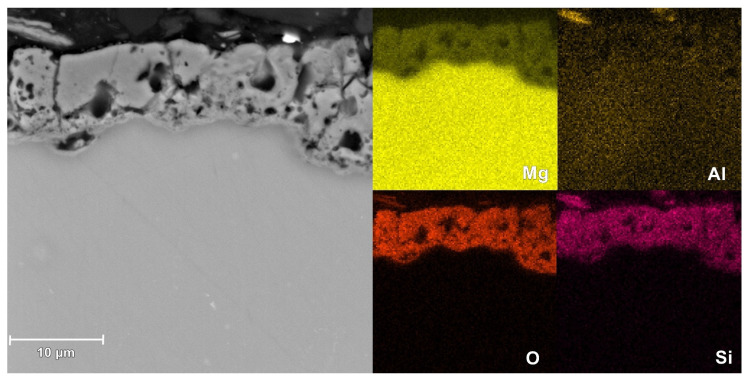
Microstructure and elemental distribution in the cross-section of the coating for the Base sample, measured by EDS.

**Figure 11:**
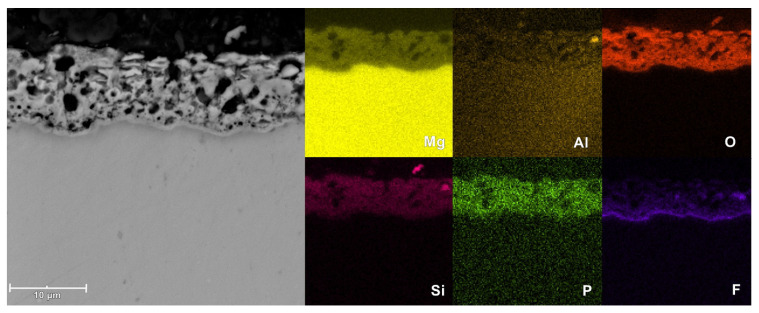
Microstructure and elemental distribution in the cross-section of the coating for the 2.5PF6 sample, measured by EDS.

**Figure 12:**
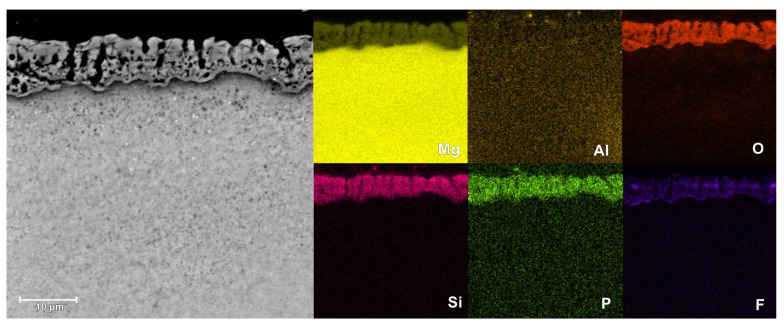
Microstructure and elemental distribution in the cross-section of the coating for the FPO4 sample, measured by EDS.

The SEM images of the surface microstructure of PEO coatings formed on AM50 alloy in different electrolytes are shown in Figure 9. The cross-sectional morphology and EDS elemental mapping of the coatings are shown in Figure 10, Figure 11 and Figure 12.

For all samples, the surface is rich in pores, which is typical for PEO coatings. It can be seen that in the case of the coating prepared with the addition of 2.5 g/L KPF_6_, the pores are smaller and distributed more evenly than in the other coatings (no large external pores). However, the morphology of this coating resembles a sponge-like structure, in contrast to the crater-like structures observed in other coatings (Figure 9b,e).

Based on the cross-sectional images, it can be concluded that in all cases the coatings show excellent adhesion to the substrate. The structure of the coatings includes an outer, thick, porous layer and an inner (localized right next to the substrate material), thin, barrier layer. The coating produced in a silicate bath (Base sample) is characterized by lower porosity of the outer layer, compared to that of other coatings. However, in this case the inner layer is thinner and less compact. In the case of the coating prepared with the addition of KPF_6_ (2.5PF6 sample), the inner layer appears to be the most continuous and without damages. This causes the conversion coating with the addition of KPF_6_ to have the best anticorrosive properties.

The EDS measurements have shown that the PEO coatings are composed of magnesium, aluminum, oxygen, silicon, and in the case of 2.5PF6 and FPO4 coatings, also phosphorus and fluorine. These elements, with the exception of fluorine, are evenly distributed in the conversion coating. In the case of fluorine, an increase in its content is visible near the magnesium substrate (in the inner layer of the PEO coating).

Using XPS analysis, the chemical composition of the outer and inner layers of the coatings obtained in the PEO process was determined, and the results are listed in Table 8.

The XPS results indicate that the main components of the coatings obtained are Mg from the substrate as well as O and Si, which are components of the basic silicate electrolyte. Small amounts of Al were also found (Al is a component of the AM50 alloy). When the electrolyte contained KPF_6_ (sample 2.5PF6) or a mixture of NaF + Na_3_PO_4_ (sample FPO4), fluorine and phosphorus were also present in the coatings. The 2.5PF6 coating contained more fluorine than the FPO4 coating, while a reverse relation was observed in the case of phosphorus. It should be noted that phosphorus was evenly incorporated into the coating, but in the case of fluorine, its clear enrichment is visible in the inner layer of the PEO coating.

**Figure 13:**
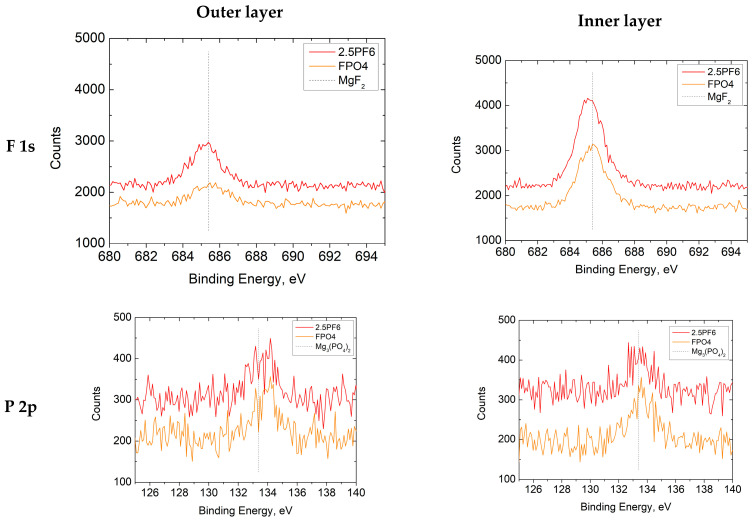
XPS high-resolution single peak spectra of the F 1s and the P 2p regions for 2.5PF6 and FPO4 PEO coatings.

High-resolution XPS analyses of the F 1s and P 2p peaks have been performed to identify chemical compounds formed in the modified PEO coatings, and the results are presented in Figure 13. The specific spectra of F 1s for both coatings (2.5PF6 and FPO4), regardless of the depth (outer/inner layer), have shown one peak at 685.1 ± 0.1 eV, which corresponds to the presence of MgF_2_ (metal fluoride at 685.0 ± 0.9 eV [57]). The P 2p spectra have also shown only one peak at 133.4 ± 0.2 eV, suggesting the presence of Mg_3_(PO_4_)_2_ {(PO_4_)^3−^ at 133.2 ± 0.7 eV [57]} in both layers. The results presented exclude the formation of Mg(PF_6_)_2_, and the binding energy values for F 1s and P 2p are higher: 687.8 ± 0.2 eV and 136.4 ± 0.8 eV, respectively [57]. This is in contrast to our previous finding that Mg(BF_4_)_2_ is present mainly in the inner layer [46]. Using DFT calculation, it has been shown that BF_4_^−^ and H_3_O^+^ form stronger hydrogen bonds than PF_6_^−^ and H_3_O^+^, which suggests that clusters formed by the former pair are more stable [58]. These findings can be considered as a reasonable explanation of the observed discrepancy between BF_4_^−^ and PF_6_^−^ behavior in PEO processes.

The compositions of the coatings obtained in the present study are in agreement with reported literature data from studies, in which conventional silicate, phosphate, and fluoride electrolytes were used. For PEO coatings synthetized in alkaline silicate baths, it has been often confirmed that they are composed of MgO and Mg_2_SiO_4_ [17,22]. In the case of phosphate baths, the most frequently reported component of coatings is Mg_3_(PO_4_)_2_ [17,24]. In the presence of simple fluoride salts (NaF, KF), the common ingredient of a coating is MgF_2_ [14,33,34]. In the case of polyfluorine salts, the hydrolysis of the complex anion to a simple fluoride ion (and further reaction with the magnesium cation to form MgF_2_) and the formation of an oxide or a salt derived from the other element are observed (e.g., ZrF_6_^2−^ to ZrO_2_ [39], TiF_6_^2−^ to TiO_2_ [41], SiF_6_^2−^ to Mg_2_SiO_4_ [42], and AlF_6_^3−^ to Al_2_O_3_ [44]). Therefore, the use of the KPF_6_ additive in the electrolyte bath does not change the qualitative composition of the bath (compared to the mixture of orthophosphate and fluoride), but has an impact on the morphology and anticorrosive properties of the coatings obtained. The mechanism of this behavior can probably be related to the hydrolysis of the PF_6_^−^ ion in the PEO process, leading to the formation of fluoride (F^−^) and phosphate (PO_4_^3−^) ions, as well as monofluorophosphate (PO_3_F^2−^), difluorophosphate (PO_2_F_2_^−^), and hydrofluoric acid (HF) [56].

Based on data from the literature and the results obtained, the following processes can be proposed, which lead to the formation of coatings on magnesium in an alkaline silicate bath containing KPF_6_.

Mg^0^ → Mg^2+^ + 2e^−^ (5)

Mg^2+^ + 2OH^−^ → Mg(OH)_2_ → MgO + H_2_O (6)

2Mg^2+^ + SiO_3_^2−^+ 2OH^−^ → Mg_2_SiO_4_ + H_2_O (7)

PF_6_^−^ + 8OH^−^ → [PO_4_]^3−^ + 6F^−^ + 4H_2_O (8)

3Mg^2+^ + 2PO_4_^3−^→ Mg_3_(PO_4_)_2_ (9)

Mg^2+^ + 2F^−^ → MgF_2_ (10)

## 4. Conclusions

In the presented study, the PEO coatings produced on the AM50 Mg alloy in alkaline silicate baths with the addition of KPF_6_ have been investigated. The results obtained can be summarized as follows:

- The anticorrosive properties of the obtained coatings increase when the KPF_6_ concentration is increased to 2.5 g/L. The addition of larger amounts of KPF_6_ causes damage to the coating (a large increase in its roughness), probably due to the local formation of HF during the PEO process.
- The addition of KPF_6_ allows for better anticorrosive properties of the synthesized coating to be obtained, compared to a mixture of NaF and Na_3_PO_4_ with an equimolar content of fluorine and phosphorus.
- XPS measurements have shown that in coatings obtained in the presence of KPF_6_, as well as a mixture of NaF and Na_3_PO_4_ in the baths, the coating components derived from these additives are the same [MgF_2_ and Mg_3_(PO_4_)_2_]. Mg(PF_6_)_2_ was not present in the formed coatings, which is in contrast to the formation of Mg(BF_4_)_2_, when the silicate bath contained NaBF_4_ [46].
- The surface morphology of the PEO coatings produced in the KPF_6_-containing baths was more uniform and showed a sponge-like structure, in contrast to commonly reported crater-like structures. The sponge-like structure is similar to bone structure, and in combination with the presence of phosphates, it can increase the biocompatibility and the possibility of self-healing of this coating.

## Figures and Tables

**Table 1:** Chemical composition (wt.%) of the AM50 alloy according to EN 1753:2019 [48].

Al	Mn	Zn	Si	Fe	Cu	Ni	Mg
4.40–5.50	min. 0.1	max. 0.02	max. 0.1	max. 0.005	max. 0.01	0.002	balance

**Table 2:** Time to reach the critical voltage (450 V) and the final voltage of the PEO processes in the electrolytes containing different concentrations of KPF_6_.

Concentration of KPF_6_, g/L	Time to Reach Critical Voltage, min	Final Voltage, V
0.0	8.8	455
0.5	8.3	461
1.0	8.0	465
1.5	7.0	466
2.0	5.9	467
2.5	5.0	470
3.0	9.5	453
4.0	-	346

**Table 3:** Electrochemical parameters of PEO coatings synthesized in electrolytes containing different concentrations of KPF_6_, obtained from polarization measurements after 3 h exposure in DHS.

Concentration of KPF_6_, g/L	*E*_CORR_, V	*j*_CORR_, µA/cm^2^	*R*_P_,kΩ∙cm^2^
0.0	−1.630	33.01	1.04
0.5	−1.606	23.11	1.46
1.0	−1.596	9.35	3.81
1.5	−1.595	4.55	7.88
2.0	−1.593	3.91	9.41
2.5	−1.587	1.76	21.09
3.0	−1.555	2.94	13.09
4.0	−1.453	5.94	7.25

**Table 4:** EIS fitting parameters for coatings obtained at different concentrations of KPF_6_.

Concen. of KPF_6_, g/L	*R*_S_, Ω·cm^2^	*Q*_OL_, µF^n^/cm^2^	*n*_OL_	*R*_OL_, Ω·cm^2^	*Q*_IL_, µF^n^/cm^2^	*n*_IL_	*R*_IL_, kΩ·cm^2^	*C*_dl_, mF/cm^2^	*R*_ct_, kΩ·cm^2^	*L* kH·cm^2^	*R*_L_ kΩ·cm^2^	*R*_total_ kΩ·cm^2^	Χ^2^
0	155.5	-	-	-	5.713	0.877	3.296	0.469	1.533	25.5	0.75	3.80	5.12 × 10^−4^
0.5	146.8	1.032	0.905	37.9	4.189	0.917	3.476	0.516	1.927	53.8	3.87	4.80	2.88 × 10^−4^
1.0	147.9	0.784	0.913	42.5	3.347	0.926	4.971	0.348	2.928	24.2	18.64	7.54	4.25 × 10^−4^
1.5	135.9	0.814	0.858	79.1	2.051	0.922	5.728	0.178	4.361	200.7	21.60	9.44	3.31 × 10^−4^
2.0	153.9	0.803	0.873	74.2	1.974	0.922	6.222	0.180	4.550	233.1	15.92	9.84	2.74 × 10^−4^
2.5	141.1	0.392	0.847	218.2	0.819	0.892	13.400	0.093	9.271	231.5	28.51	20.61	5.13 × 10^−4^
3.0	151.1	0.425	0.867	129.4	1.431	0.894	7.921	0.189	7.069	293.1	16.92	13.04	3.30 × 10^−4^
4.0	144.9	1.115	0.896	64.9	2.818	0.903	5.975	0.345	5.035	-	-	11.07	6.37 × 10^−4^

**Table 5:** Composition of electrolytes used in comparative studies of PEO coatings.

Sample	Composition of Electrolyte, g/L	Molar Contents of Fluorine, mM/L	Molar Contents of Phosphorus, mM/L
Base	Na_2_SiO_3_·5H_2_O: 10	-	-
	NaOH: 4		
2.5PF6	Na_2_SiO_3_·5H_2_O: 10	81.5	13.6
	NaOH: 4		
	KPF_6_: 2.5		
FPO4	Na_2_SiO_3_·5H_2_O: 10	81.5	13.6
	NaOH: 4		
	NaF: 3.42		
	Na_3_PO_4_·12H_2_O: 5.17		

**Table 6:** Electrochemical parameters of the uncoated AM50 alloy and the PEO coatings synthesized in different electrolytes from the polarization measurements after 3 h exposure in DHS.

Sample	*E*_CORR_, V	*j*_CORR_, µA/cm^2^	*R*_P_,kΩ∙cm^2^
Uncoated AM50	−1.607	140.10	0.22
Base	−1.630	33.01	1.04
2.5PF6	−1.587	1.76	21.09
FPO4	−1.501	2.75	13.41

**Table 7:** EIS for the coatings obtained from different electrolytes.

Sample	*R*_S_, Ω·cm^2^	*Q*_OL_, µF^n^/cm^2^	*n*_OL_	*R*_OL_, Ω·cm^2^	*Q*_IL_, µF^n^/cm^2^	*n*_IL_	*R*_IL_, kΩ·cm^2^	*C*_dl_, mF/cm^2^	*R*_ct_, kΩ·cm^2^	*L* kH·cm^2^	*R*_L_ kΩ·cm^2^	*R*_total_ kΩ·cm^2^	Χ^2^
Base	155.5	-	-	-	5.713	0.877	3.296	0.469	1.533	25.5	0.75	3.80	5.12 × 10^−4^
2.5PF6	141.1	0.392	0.847	218.2	0.819	0.892	13.400	0.093	9.271	231.5	28.51	20.61	5.13 × 10^−4^
FPO4	130.6	0.828	0.890	63.7	2.411	0.910	10.270	0.016	5.271	-	-	15.60	5.73 × 10^−4^
**Sample**	***R*** **_S_** **,** **Ω·cm^2^**				***Q*** **_C_** **,** **µF^n^/cm^2^**	***n*** **_C_**	***R*** **_C_** **,** **k** **Ω·cm^2^**	***C*** **_dl_** **,** **mF/cm^2^**	***R*** **_ct_** **,** **k** **Ω·cm^2^**	***L*** **kH** **·cm^2^**	***R*** **_L_** **k** **Ω·cm^2^**	***R*** **_total_** **k** **Ω·cm^2^**	**Χ** **^2^**
Uncoated AM50	122.9				0.302	0.873	0.073	7.627	0.029	1.53	0.153	0.061	2.50 × 10^−4^

**Table 8:** Elemental contents (in atomic percentage) determined by XPS for the coatings obtained in different electrolytes.

Sample	Layer	Elements Content, at.%					
		Mg	O	Si	Al	F	P
Base	outer	49.1	37.3	11.2	2.4	-	-
	inner	53.8	37.4	6.4	2.5	-	-
2.5PF6	outer	39.6	43.0	10.1	3.7	2.6	1.1
	inner	39.6	40.5	9.1	3.3	6.3	1.1
FPO4	outer	45.0	41.3	7.6	3.1	1.7	1.4
	inner	38.9	42.8	7.4	3.7	5.2	1.9

## Data Availability

Data available on request from the authors.
